# Rcs phosphorelay affects the sensitivity of *Escherichia coli* to plantaricin BM-1 by regulating biofilm formation

**DOI:** 10.3389/fmicb.2022.1071351

**Published:** 2022-11-24

**Authors:** Zheng Bian, Wenbo Liu, Junhua Jin, Yanling Hao, Linshu Jiang, Yuanhong Xie, Hongxing Zhang

**Affiliations:** ^1^Beijing Laboratory of Food Quality and Safety, Beijing Key Laboratory of Agricultural Product Detection and Control of Spoilage Organisms and Pesticide Residue, Beijing Engineering Technology Research Center of Food Safety Immune Rapid Detection, College of Food Science and Engineering, Beijing University of Agriculture, Beijing, China; ^2^Department of Nutrition and Health, Key Laboratory of Functional Dairy, Co-constructed by Ministry of Education and Beijing Government, China Agricultural University, Beijing, China; ^3^Animal Science and Technology College, Beijing University of Agriculture, Beijing, China

**Keywords:** bacteriocins, Rcs Phosphorelay, proteome, biofilm, *Escherichia coli*

## Abstract

**Introduction:** Plantaricin BM-1 is a class IIa bacteriocin produced by *Lactobacillus plantarum* BM-1 that exerts significant antibacterial activity against many foodborne bacteria. Studies have shown that class IIa bacteriocins inhibit Gram-positive bacteria *via* the mannose phosphotransferase system; however, their mechanism of action against Gram-negative bacteria remains unknown. In this study, we explored the mechanism through which the Rcs phosphorelay affects the sensitivity of *Escherichia coli* K12 cells to plantaricin BM-1.

**Methods and Results:** The minimum inhibitory concentrations of plantaricin BM-1 against *E. coli* K12, *E. coli* JW5917 (*rcsC* mutant), *E. coli* JW2204 (*rcsD* mutant), and *E. coli* JW2205 (*rcsB* mutant) were 1.25, 0.59, 1.31, and 1.22 mg/ml, respectively. Growth curves showed that *E. coli* JW5917 sensitivity to plantaricin BM-1 increased to the same level as that of *E. coli* K12 after complementation. Meanwhile, scanning electron microscopy and transmission electron microscopy revealed that, under the action of plantaricin BM-1, the appearance of *E. coli* JW5917 cells did not significantly differ from that of *E. coli* K12 cells; however, cell contents were significantly reduced and plasmolysis and shrinkage were observed at both ends. Crystal violet staining and laser scanning confocal microscopy showed that biofilm formation was significantly reduced after *rcsC* mutation, while proteomic analysis identified 382 upregulated and 260 downregulated proteins in *E. coli* JW5917. In particular, *rcsC* mutation was found to affect the expression of proteins related to biofilm formation, with growth curve assays showing that the deletion of these proteins increased *E. coli* sensitivity to plantaricin BM-1.

**Discussion:** Consequently, we speculated that the Rcs phosphorelay may regulate the sensitivity of *E. coli* to plantaricin BM-1 by affecting biofilm formation. This finding of class IIa bacteriocin against Gram-negative bacteria mechanism provides new insights.

## Introduction

Bacteriocins are a class of peptides or proteins that are synthesized by ribosomes and usually exert bactericidal activity against species which are closely related to the producer bacterium (Klaenhammer, 1988; Simons et al., 2020). In recent years, an increasing number of bacteriocins have been used for food production and storage, and several bacteriocins from lactic acid bacteria (LAB) have shown good bacteriostatic activity against foodborne bacteria (Zhang et al., 2022). Pediocin PA-1 is a bacteriocin produced by LAB that exhibits extremely strong bacteriostatic activity against *Listeria monocytogenes* and is considered to be a natural food biological preservative due to its high bacteriostatic activity and low toxicity (Rodriguez et al., 2002).

Known bacteriocins can be divided into three main categories according to their heat resistance and size: class I, class II, and class III (Alvarez-Sieiro et al., 2016). Class II bacteriocins are small, thermostable, non-modified, and less than 10 kDa in size. Class IIa bacteriocins are broad-spectrum antibacterial agents of 36–49 amino acids in length that are particularly effective against *L. monocytogenes* and typically consist of two domains (Fregeau Gallagher et al., 1997). The first domain contains a highly conserved cationic N-terminal region of cations, with the amino acid sequence, YGNGV/L, whereas the second domain includes the poorly conserved C-terminal region with a hairpin or functionally equivalent helix-hinge-helix structures (Kjos et al., 2011). Previous studies have suggested that the class IIa bacteriocins target receptor in Gram-positive bacteria is the sugar transporter mannose phosphotransferase system (Man-PTS), which is comprised four components: IIA, IIB, IIC, and IID. Studies have demonstrated that only the IIC and IID membrane localization components are required for bacteriocin sensitivity (Diep et al., 2007). However, it has also been reported that class IIa bacteriocins do not target Man-PTS in Gram-negative bacteria. Therefore, the role of class IIa bacteriocin in Gram-negative bacteria mechanism requires further exploration.

Two-component systems (TCS) is versatile transmembrane signaling solutions that typically consist of a membrane-embedded sensor histidine kinase (HK) and a cytoplasmic response regulator (RR; Delhaye et al., 2019). The sensor HK responds to environmental signals and converts external stimuli into adaptive signals through the autophosphorylation of conserved histidine residues. The phosphate group bound by the HK histidine residue is subsequently transferred to a specific aspartate residue on the cognate RR for activation *via* a phosphotransfer reaction during which unphosphorylated HK acts as a phosphatase by removing phosphoryl groups from RR, thus maintaining a balance between active and inactive states (West and Stock, 2001; Logre et al., 2020). The His-Asp-His-Asp phosphorelay is a more complicated version of two-component (West and Stock, 2001). Unlike a typical HK-RR two-component system, the Rcs phosphorelay has three core components: RcsC (HK), RcsB (RR), and RcsD (an intermediate inner membrane phosphorelay protein; Cho et al., 2014). The Rcs phosphorelay controls the expression of several genes, including periplasmic quality control, biofilm formation, toxicity, and motility (Majdalani and Gottesman, 2005; Clarke, 2010), and has been reported to protect *Escherichia coli* from TseH toxicity by regulating mechanisms such as capsular synthesis (Hersch et al., 2020).

*Lactobacillus plantarum* BM-1 isolated from traditionally fermented Chinese meat products can produce plantaricin BM-1, a novel class IIa bacteriocin that exerts significant bacteriostatic activity against many foodborne bacteria (Zhang et al., 2013). Previously, we found that the loss of the BasS/BasR TCS affects the sensitivity of *E. coli* K12 to plantaricin BM-1 and this loss affects the PhoQ-PhoP, BasS-BasR, and Rcs phosphorelay regulatory networks (Liu et al., 2022). However, the effects of Rcs phosphorelay in the sensitivity of *E. coli* to bacteriocins remain unknown. Here, we investigated the regulatory role of the Rcs phosphorelay in the sensitivity of *E. coli* to plantaricin BM-1.

## Materials and methods

### Preparation of plantaricin BM-1

Plantaricin BM-1 was prepared as described previously (Zhang et al., 2013). *Lactobacillus plantarum* BM-1 was cultured in de Man, Rogosa, and Sharpe (MRS) broth at 37°C for 12 h. The supernatant was collected by centrifugation (4°C, 10,000 rpm) and stirred overnight at 4°C for ammonium sulfate precipitation. After the precipitate had been solubilized, plantaricin BM-1 was purified using dialysis, desalting, and cation exchange before being freeze-dried. The freeze-dried powder was redissolved in 0.22 μm filtration membrane and stored at −80°C.

### Strains and culture conditions

The bacterial strains used in this study are listed in Table 1. *Escherichia coli* strains and *L. plantarum* BM-1 were cultured at 37°C with aeration at 180 rpm, Luria-Bertani (LB) broth and MRS broth were used, respectively.

**Table 1:** Strains used in this study.

Strains and plasmids	Characteristics	Source
*Escherichia coli* K12	Wild-type *E. coli* strain BW25113	Laboratory preservation
*E. coli* JW5917	*Escherichia coli* BW25113 with *rcsC* deletion	Keio collection
*E. coli* JW2204	*E. coli* BW25113 with *rcsD* deletion	Keio collection
*E. coli* JW2205	*E. coli* BW25113 with *rcsB* deletion	Keio collection
*L. plantarum* BM-1	*Lactobacillus plantarum* BM-1, producing plantaricin BM-1	Laboratory preservation
pKD46	Plasmid containing the lambda Red system, L-arabinose inducible	BioVector NTCC
*E. coli* ReJW5917	*E. coli* JW5917 with *rcsC* complemented	This study
*E. coli* JW5431	*E. coli* BW25113 with *gutQ* deletion	Keio collection
*E. coli* JW0820	*E. coli* BW25113 with *bssR* deletion	Keio collection
*E. coli* JW1504	*E. coli* BW25113 with *lsrK* deletion	Keio collection
*E. coli* JW0389	*E. coli* BW25113 with *phoB* deletion	Keio collection
*E. coli* JW2366	*E. coli* BW25113 with *evgA* deletion	Keio collection
*E. coli* JW2367	*E. coli* BW25113 with *evgS* deletion	Keio collection
*E. coli* JW5437	*E. coli* BW25113 with *rpoS* deletion	Keio collection

### Minimal inhibitory concentration determination

Minimal inhibitory concentration (MIC) values were determined as described previously, with some modifications (Chen et al., 2021). Briefly, *E. coli* K12 was pre-cultured in LB broth for 12 h until log phase, diluted to 10^4^ CFU/ml, and added to 96-well plates at 100 μl per well. Plantaricin BM-1 was quantified using a NanoDrop 2000 (Thermo Fisher Scientific, Shanghai, China), diluted two-fold, and added to a 96-well plate containing 100 μl of *E. coli* K12 per well. After mixing, the 96-well plate was incubated at 37°C for 12 h and the optical density (OD) at 600 nm (OD600) was measured using an enzyme-linked immunosorbent assay (ELISA) plate reader (ELX808, BioTek, VT, United States). The lowest concentration of plantaricin BM-1 able to inhibit the growth of *E. coli* K12, JW5917, JW2204, and JW2205 (i.e., no increase in OD600) was recorded as the MIC. Each experiment was performed in triplicate.

### Construction of an *rcsC*-complemented mutant of mutant of *Escherichia coli* JW5917

Complementary *rcsC* mutants of *E. coli* JW5917 were performed according to the method of Juhas and Ajioka (2016). Briefly, pKD46 plasmids were transformed into competent *E. coli* JW5917 using 0.1 mol/ml cold CaCl_2_ and homologous recombinase expression was induced by incubation with 0.50 mg/ml L-arabinose in LB broth at 30°C. The following primers were used to amplify *rcsC* gene fragment from *E. coli* K12: *rcsC*-F (5′-3′) TGA GGC GGA GCT TCG CCC CTG TTA GTG CTC TGG CTG TTG and *rcsC*-R (5′-3′) CGC ATT TGC GGA ATA GGC AGA ATC TGC GAT GAT GAA GC (homology underlined). After transfection into competent *E. coli* JW5917 cells, the cells were incubated in 900 μl of LB broth at 37°C for 2 h, diluted with saline, plated on LB agar, and incubated at 37°C for 12 h. Single recombinant ReJW5917 colonies were verified using *rcsC*-F/R primers.

### Bacterial growth assays

Wild-type *E. coli* K12, JW5917, JW2204, JW2205, and ReJW5917 (3.00 log_10_ CFU/mL) were cultured in LB broth with or without plantaricin BM-1 (2× MIC of *E. coli* K12) at 37°C for 12 h. Bacterial suspensions were collected every 2 h, cells were performed using plate colony counting method (Masuda and Tomioka, 1978), and the average results of the three experiments were plotted. All experiments consisted of three replications.

### Electron microscopy

#### Scanning electron microscopy

Wild-type *E. coli* K12 and JW5917 (3.00 log_10_ CFU/mL) were cultured in LB broth at 37°C for 12 h with or without plantaricin BM-1 (2× MIC of *E. coli* K12). The sample processing method has been modified based on the method of Luo et al. (2021). The bacterial suspension was then centrifuged at 6,000 ×*g* for 10 min. After the supernatant was removed, the cells were cleaned three times with 0.10 M phosphate-buffered brine (PBS) buffer (pH 7.2) to remove the residual BM-1, fixation solution (2.50% glutaraldehyde) was added, and the cells were fixed overnight. After washing with PBS for 4 times within 20 min, the cells were gradually dehydrated with different concentrations of ethanol, and then replaced in replacement solution (100% acetone) for 20 min. Then washed with 100% tert-butanol for 3 times and freeze-dried for 2 h. Finally, the cells were electrically treated and sprayed with 80 nm gold powder, and imaged using Scanning electron microscopy (SEM; SU8100, Hitachi, Japan).

#### Transmission electron microscopy

Prior to Transmission electron microscopy (TEM), cell strains were pretreated using the same methods as for SEM. Cell samples embedded in white resin capsules were then cut into ultra-thin slices (50–90 nm). The sections were then fixed on copper mesh and stained with lead citrate and uranyl acetate, respectively. After drying, the sections were observed by TEM HT7800 (Hitachi, Japan).

### Biofilm determination

#### Crystal violet staining assay

To determine the regulatory mechanism of *rcsC* mutants on plantaricin BM-1 and biofilm formation, the experimental method of crystal violet staining is modified on the experimental method of Luo et al. (2021). *E. coli* K12, JW5917, and ReJW5917 were grown to log phase in LB broth, cells were centrifuged at 4,000 ×*g* for 15 min at 4°C and serially diluted to 3.00 log_10_ CFU/ml. A 100 μl aliquot of the bacterial suspension was added to a 96-well plate with fresh LB broth as a negative control. After incubation at 37°C for 24 h, the unadsorbed *E. coli* was discarded and the 96-well plates were rinsed with PBS for 3 times. Next, 200 μl of methanol solution was added to each well to fix the biofilm for 15 min at room temperature and the methanol was carefully aspirated. After the plate had been dried at room temperature, added 200 μl of 0.1% crystal violet and incubated for 15 min at room temperature, then excess crystal violet was removed by washing the cells with PBS. After the plate had been dried at room temperature, added 200 μl aliquot of 33% glacial acetic acid and incubated at 37°C for 30 min. The absorbance at 590 nm was measured using an ELISA plate reader (ELX808, BioTek, VT, United States). The experiments were repeated three times, with five replicates per group.

#### Laser scanning confocal microscopy

To verify the results of the crystal violet experiments and confirm the effects of RcsC mutation on biofilm formation, Laser scanning confocal microscopy (LSCM) experiments were performed. *E. coli* K12, JW5917, and ReJW5917 were grown to log phase in LB broth and serially diluted to 3.00 log_10_ CFU/ml. After the strains had been cultured in LB broth for 24 h on confocal dishes, they were gently washed with PBS three times and stained with 4′,6-diamidino-2-phenylindole (DAPI) at a final concentration of 0.20 μg/ml. Excess dye was removed by washing with PBS after shaking at room temperature for 30 min. Cells were observed using a LSM880 Airyscan (Carl Zeiss, Oberkochen, Germany).

### Proteomic analysis

To screen for differentially expressed proteins between wild-type *E. coli* K12 and mutant strains, quantitative proteomic analysis was performed using a 4D label-free. *E. coli* K12 and *E. coli* JW5917 (3.00 log_10_ CFU/ml) were incubated in LB broth at 37°C for 12 h without plantaricin BM-1. After centrifuge, bacterial samples were collected and frozen in liquid nitrogen. The samples were placed on ice in the frozen state and treated with protein cracking buffer (8 M urea, 1% sodium dodecyl sulfate, protease inhibitor). The supernatant of the protein was obtained by ultrasound on the ice for 2 min, cracking for 30 min, and centrifugation for 30 min at 12,000 ×*g*. The concentration of the extracted protein was determined using the Pierce BCA protein assay kit (No.23225, Thermo Fisher Scientific, MA, United States) and verified using Sodium dodecyl sulfate-polyacrylamide gel electrophoresis (SDS-PAGE). Iodoacetamide was used for reductive alkylation of protein samples that met the standard, and an equal amount of protein was taken from each sample for Trypsin/P trypsin digestion. Each sample was separated using a ultra-performance liquid chromatography (UPLC) NanoElute system (Bruker Corporation, MA, United States) with a nanoliter flow rate of buffer A (0.10% formic acid aqueous solution) and buffer B (0.10% formate acetonitrile solution). Nanoscale high-performance liquid chromatography (HPLC)-separated samples were subjected to data-dependent acquisition (DDA) mass spectrometry using a timsTOF Pro mass spectrometer (Bruker Corporation). Three biological replicates were used per sample. Liquid chromatography–tandem mass spectrometry data were matched using MaxQuant 2.0.3.1 software and the UniProt-taxonomy *E. coli* (strain K12) [83333] unique.fasta database with the results filtering parameter was Peptide FDR ≤ 0.01. Only contains at least one unique peptide protein were quantified. Differentially expressed proteins were identified based on a fold change of >1.20 or <0.83 between treatments and *p* < 0.05. Gene ontology (GO) and Kyoto Encyclopedia of Genes and Genomes (KEGG) pathway enrichment analyses were used to identify the functional subclasses and metabolic pathways related to the differentially expressed proteins.

### Sensitivity analysis of biofilm-related genes regulated by RcsC mutation to plantaricin BM-1

*Escherichia coli* JW5431, JW0820, JW1504, JW0389, JW5689, JW0665, and JW5437 strains with an initial concentration of 3.00 log10 CFU/ml were grown in LB broth at 37°C for 12 h with or without plantaricin BM-1 (2 × MIC for *E. coli* K12). Bacterial suspensions were collected every 2 h, cells were performed using plate colony counting method, and the average results of the three experiments were plotted. All experiments consisted of three replications.

### Statistical analysis method

The statistical analysis method was consistent with that used in previous studies of our laboratory. All experiments were performed in triplicate, data were presented as the mean ± SD, and the analysis of variance was used to compare viable cell counts between the growth curves of *E. coli* treated with and without plantaricin BM-1 at a significance level of 0.05 (Liu et al., 2022).

## Results

### Plantaricin BM-1 MIC determination

We determined the MIC of plantaricin BM-1 in different *E. coli* strains by culturing the strains with different concentrations of plantaricin BM-1 at 37°C for 12 h and measuring the OD600. The MIC values of plantaricin BM-1 against *E. coli* K12, JW5917, JW2204, and JW2205 were 1.25, 0.59, 1.31, and 1.22 mg/ml, respectively. We found that *E. coli* JW5917 (*rcsC* mutant) were significantly less sensitive to plantaricin BM-1 than wild-type *E. coli*.

### Construction and confirmation of *rcsC*-complementary strains

**Figure 1:**
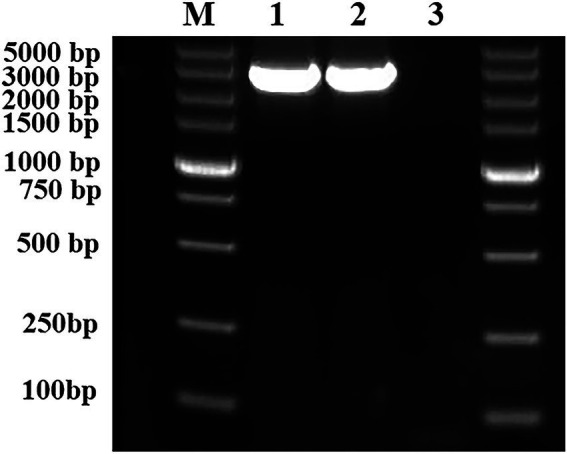
Validation of the structure of *Escherichia coli* ReJW5917. PCR products were detected using 2% agarose gel electrophoresis. Lane M represents the 5,000 bp DNA marker. Lane 1 contains the PCR product from *E. coli* K12. Lane 2 contains the PCR product from *E. coli* ReJW5917. Lane 3 contains the PCR product from *E. coli* JW5917.

To construct an *rcsC*-complementary *E. coli* JW5917 strain, a 3,076 bp *rcsC* gene fragment was amplified from the *E. coli* K12 genome using PCR with the *rcsC*-F/R primer pair and then the kanamycin resistance gene in *E. coli* JW5917 was replaced by red homologous recombination. Successful complementation was confirmed through PCR analysis of genomic DNA extracted from *E. coli* K12, *E. coli* JW5917, and the complementary *E. coli* ReJW5917 strain using *rcsC*-F/R primers. A 3,076 bp product was amplified from both *E. coli* K12 and the complementary ReJW5917 strain, but no amplified band was detected in *E. coli* JW5917 (Figure 1). Sequencing results revealed that the same *rcsC* gene fragment was amplified from *E. coli* ReJW5917 and *E. coli* K12, proving that *E. coli* ReJW5917 had been constructed successfully.

### Effect of plantaricin BM-1 on the growth of *Escherichia coli*

**Figure 2:**
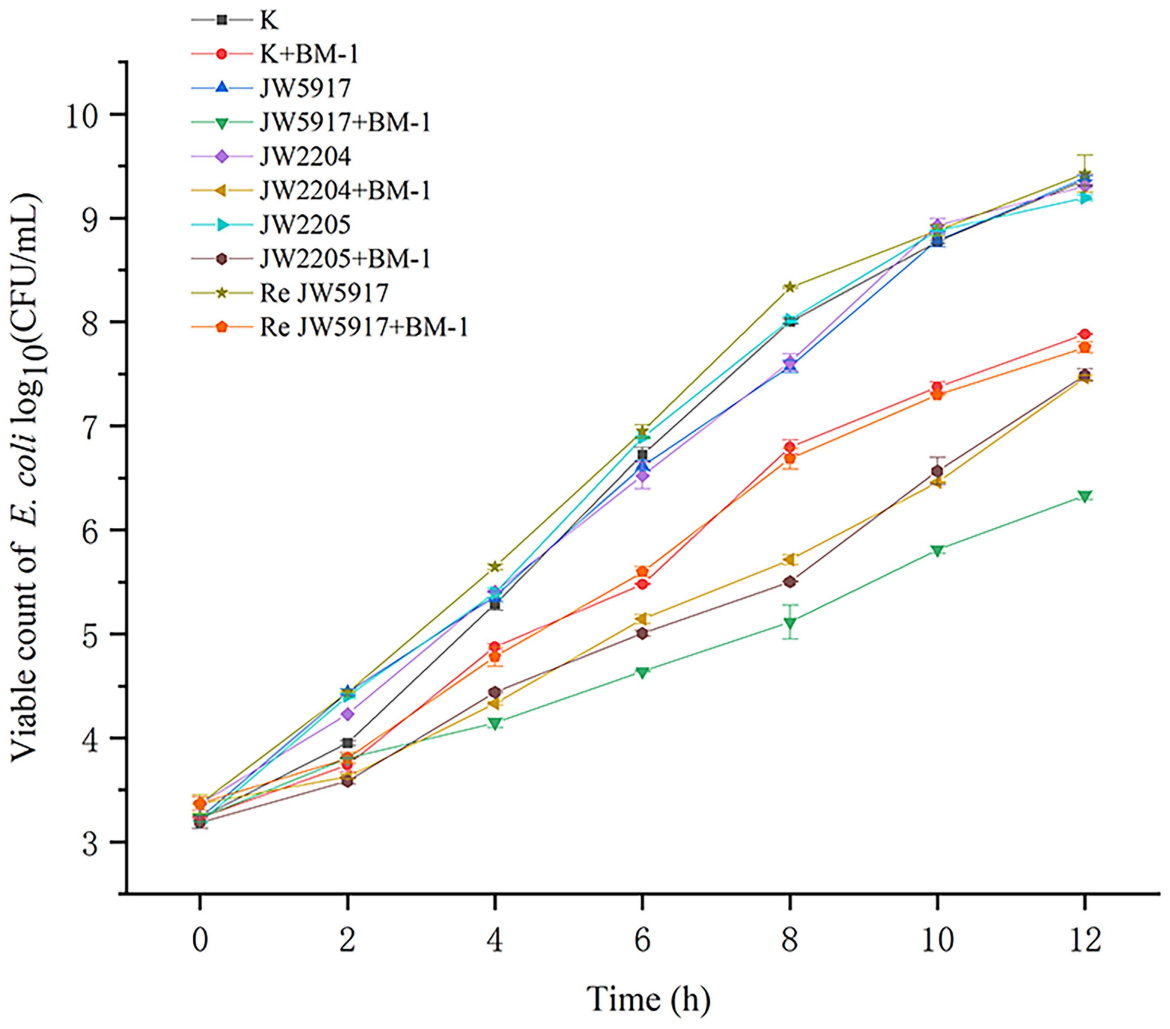
Effects of plantaricin BM-1 on the growth of wild-type *E. coli* K12 and mutant *E. coli* JW5917, JW2204, JW2205, and ReJW5917 (*p* < 0.05).

To determine the effect of plantaricin BM-1 on *E. coli* K12, JW5917, JW2204, JW2205, and ReJW5917 strains growth, growth curves were measured with and without plantaricin BM-1 treatment (Figure 2). No significant differences in growth rate were observed for *E. coli* K12, JW5917, JW2204, and JW2205, which reached similar growth levels of 9.36, 9.38, 9.30, and 9.19 log10 CFU/ml, respectively, within 12 h. After plantaricin BM-1 was treated, all strains showed slow growth, and the number of viable bacteria decreased at 12 h. The viable count of wild-type *E. coli* K12 at 12 h was 7.88 log_10_ CFU/ml, indicating relatively low sensitivity to plantaricin BM-1. Although *E. coli* JW5917 and K12 had the same growth rates at 0–2 h, *E. coli* JW5917 had a significantly lower growth rate after 2 h and the number of viable bacteria after 12 h was only 6.33 log_10_ CFU/ml, which was significantly lower than that of *E. coli* K12 (*p* < 0.05), indicating relatively high sensitivity to plantaricin BM-1. *E. coli* JW2204 and JW2205 had slightly lower growth rates than *E. coli* K12, but their viable counts at 12 h did not differ significantly compared to *E. coli* K12 (7.46 and 7.48 log_10_ CFU/ml, respectively). The growth curves of *E. coli* ReJW5917 and *E. coli* K12 were closely resembled, indicating that their susceptibility had recovered to the same level as wild-type strains. Together, these findings indicate that only the RcsC mutation in the Rcs phosphorelay affects the sensitivity of *E. coli* to plantaricin BM-1.

### Effect and comparison of plantaricin BM-1 on morphology of *Escherichia coli* K12 and JW5917

**Figure 3:**
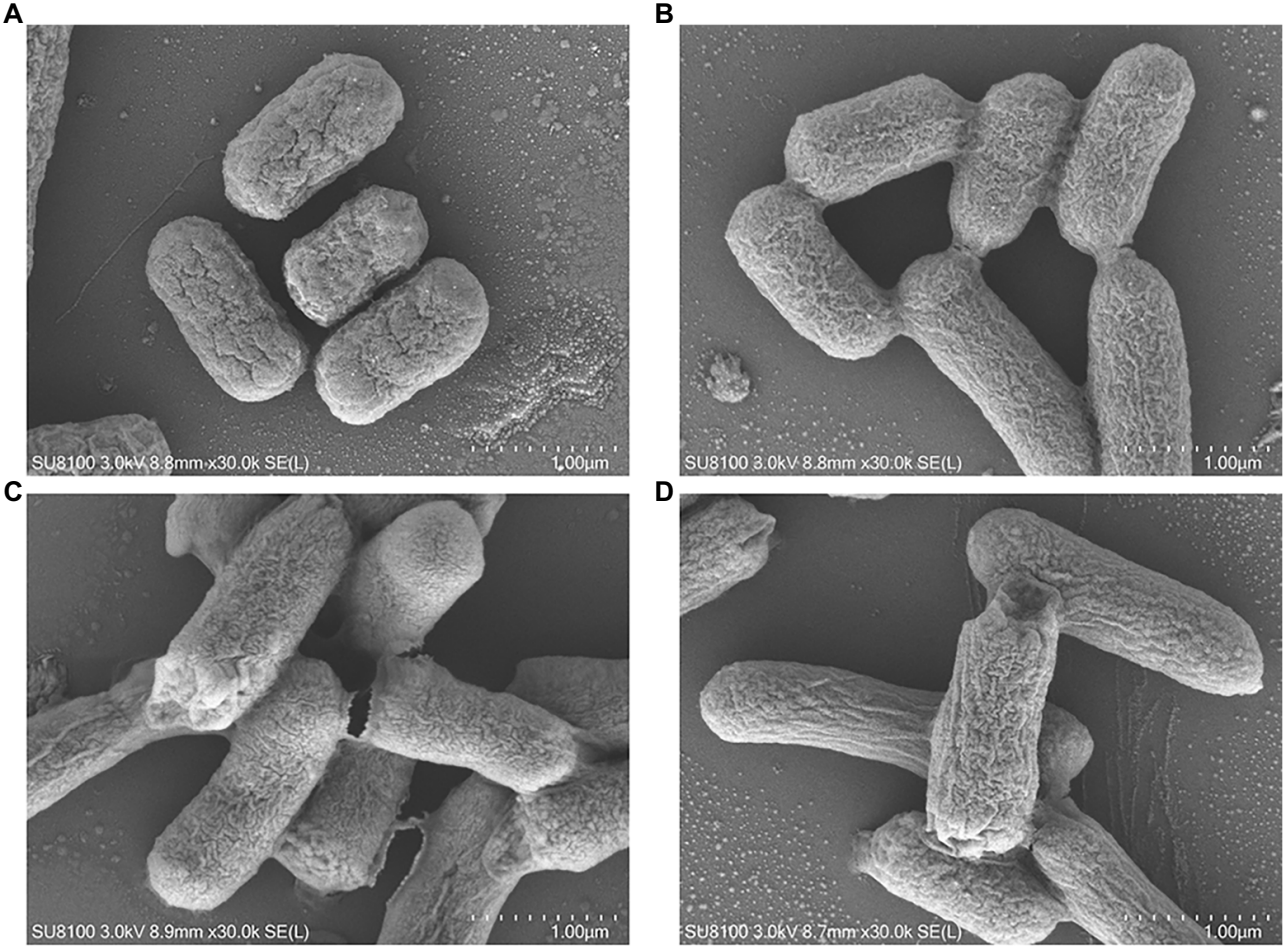
SEM images of *Escherichia coli* K12 and JW5917 strains treated with **(C,D)** and without **(A,B)** plantaricin BM-1 (magnification: 30,000×).

**Figure 4:**
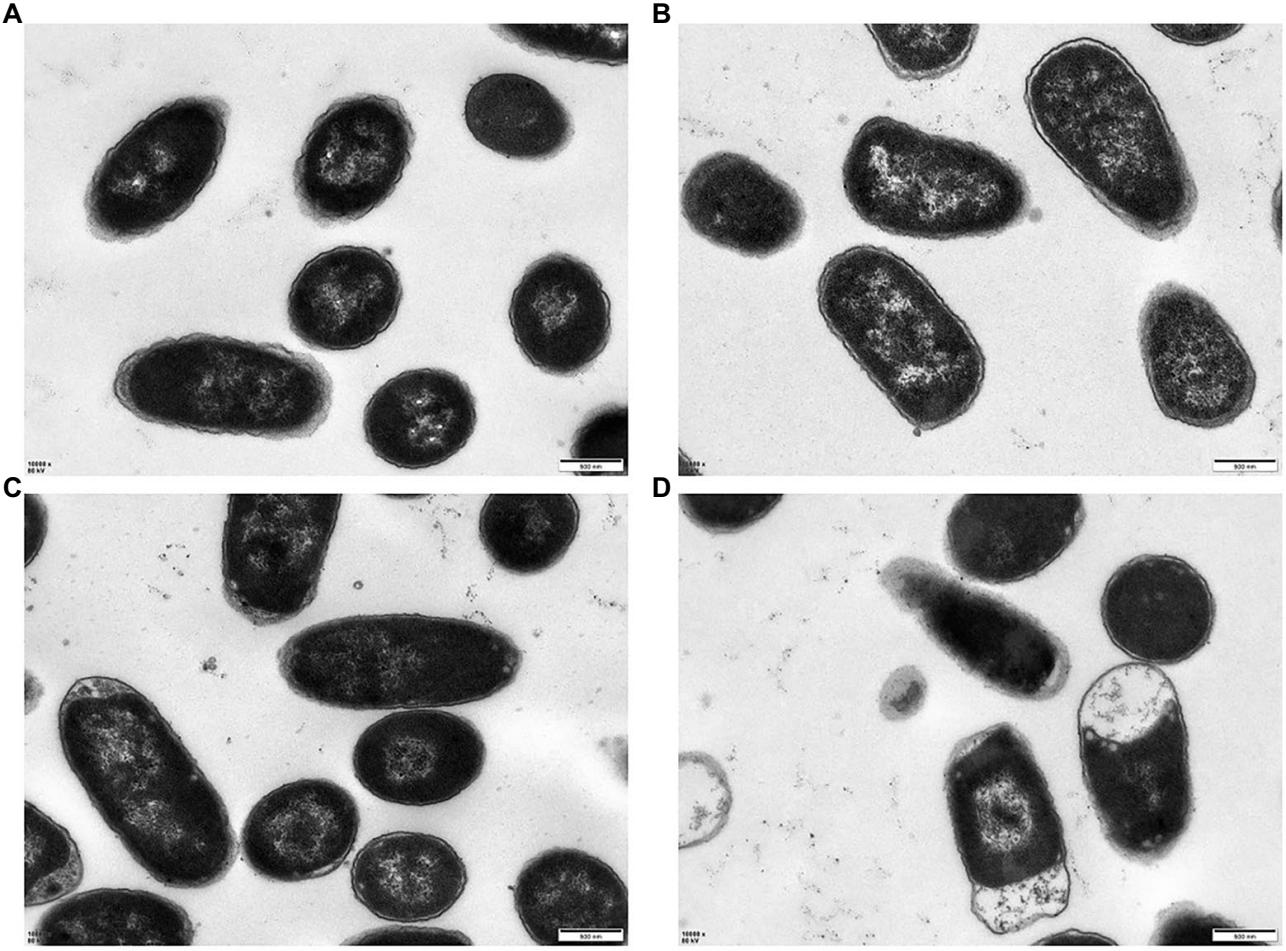
TEM images of *Escherichia coli* K12 and JW5917 treated with **(C,D)** and without **(A,B)** plantaricin BM-1 (magnification: 10,000×).

The morphological changes of *E. coli* K12 and JW5917 before and after treatment with *p*lantaricin BM-1 were observed by SEM (Figure 3) and TEM (Figure 4). In the absence of plantaricin BM-1, SEM revealed no significant differences in morphology between *E. coli* JW5917 and K12, which both had short, rod-shaped cells with smooth surfaces (Figures 3A,B). After treatment with plantaricin BM-1, both *E. coli* JW5917 and K12 showed some small changes, with individual cells becoming more folded and concave at both ends, but with no significant difference in cell morphology between the two strains (Figures 3C,D). TEM showed that in the absence of plantaricin BM-1, *E. coli* K12 cells were uniform and full, whereas *E. coli* JW5917 cell content was slightly decreased (Figures 4A,B). After plantaricin BM-1 was treated, the content of *E. coli* K12 cells did not change significantly, but individual cells showed slight shrinkage at both ends, whereas the content of *E. coli* JW5917 cells was significantly reduced and cells displayed obvious plasmolysis and shrinkage at both ends (Figures 4C,D). Therefore, the deletion of *rcsC* gene can reduce the resistance of *E. coli* K12 to plantaricin BM-1.

### Effect of *rcsC* mutant JW5917 on biofilm formation

**Figure 5:**
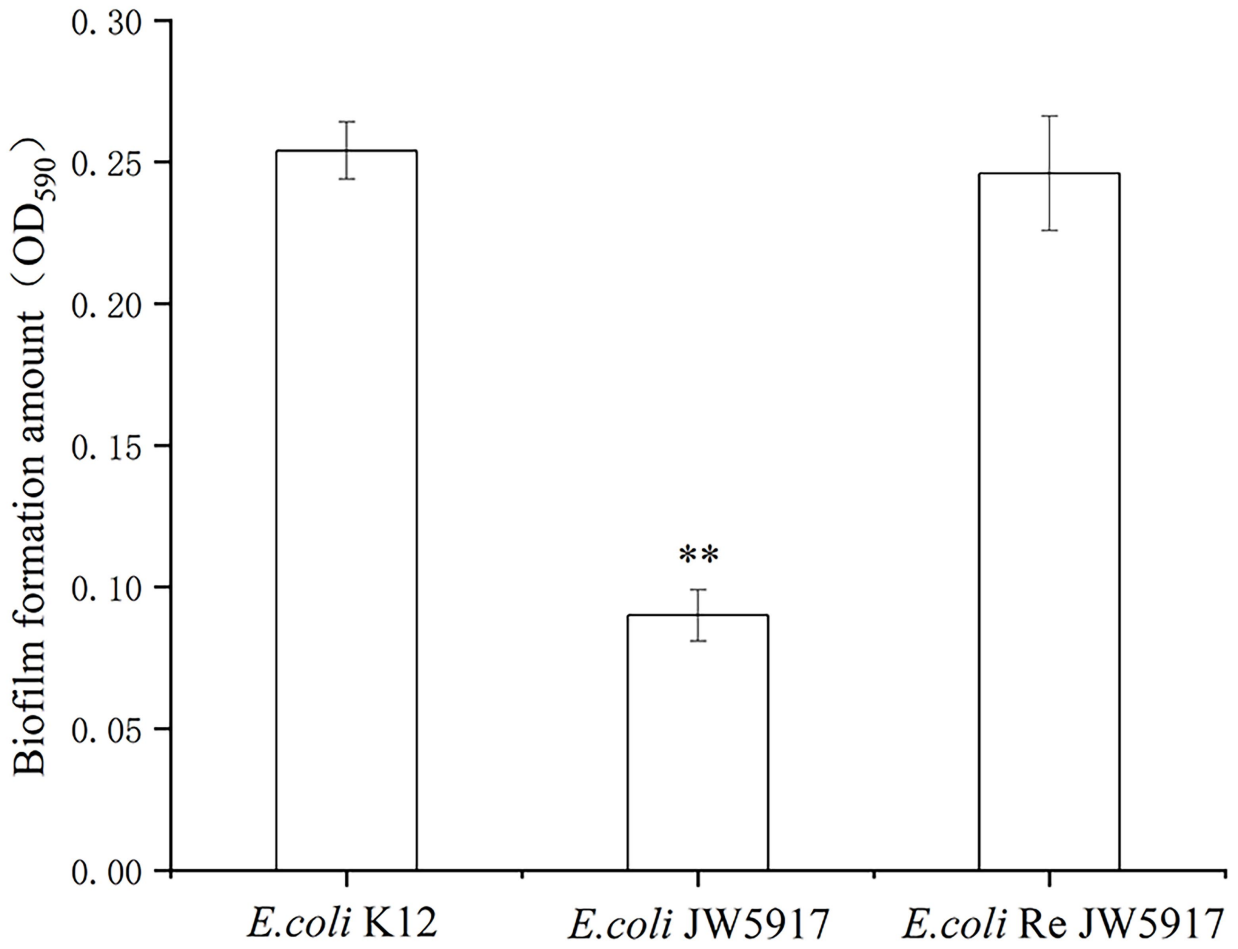
*Escherichia coli* K12, *E. coli* JW5917, and *E. coli* ReJW5917 biofilm formation. Biofilm formation detected by absorbance at 590 nm using crystal violet staining. ^**^, significance at *p* ≤ 0.01.

**Figure 6:**
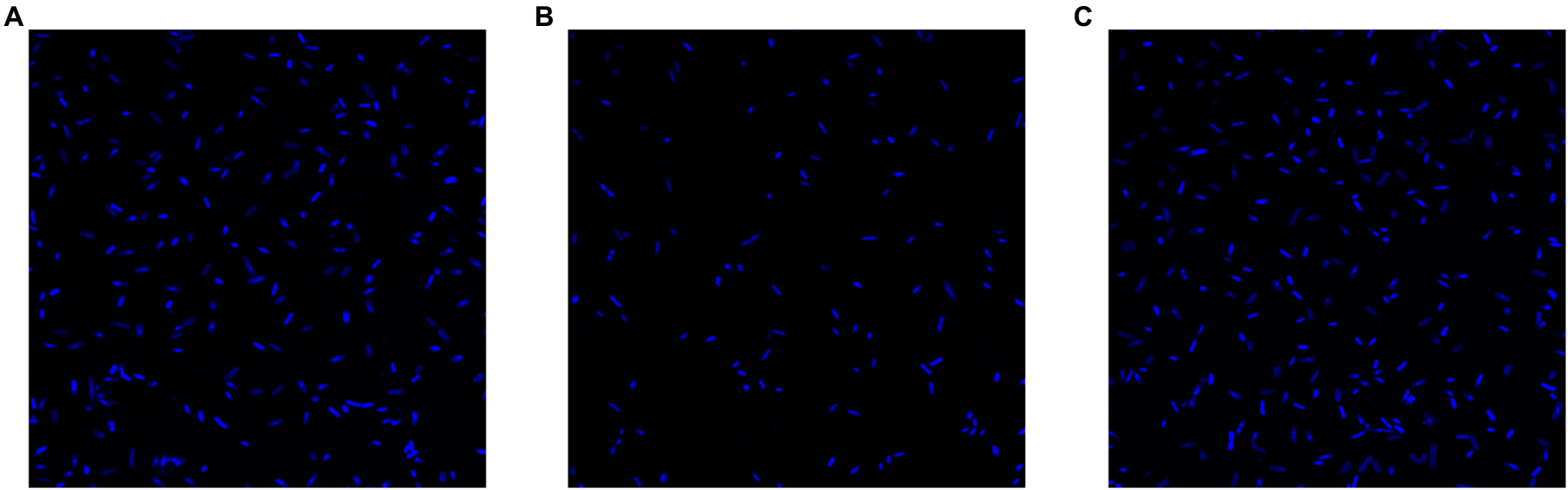
LSCM of *Escherichia coli* K12, *E. coli* JW5917, and *E. coli* ReJW5917. **(A–C)** represent *E. coli* K12, *E. coli* JW5917, and *E. coli* ReJW5917, respectively.

To evaluate the effect of the *rcsC* mutant on *E. coli* biofilm formation, we performed crystal violet staining. As shown in Figure 5, biofilm formation was significantly reduced in *E. coli* JW5917 by over 50%, whereas biofilm formation in *E. coli* ReJW5917 recovered to the same level as in *E. coli* K12. These findings were confirmed using LSCM (Figure 6), which verified that the biofilm content of *E. coli* JW5917 was significantly lower than that of *E. coli* K12 and ReJW5917.

### Proteomic analysis

**Figure 7:**
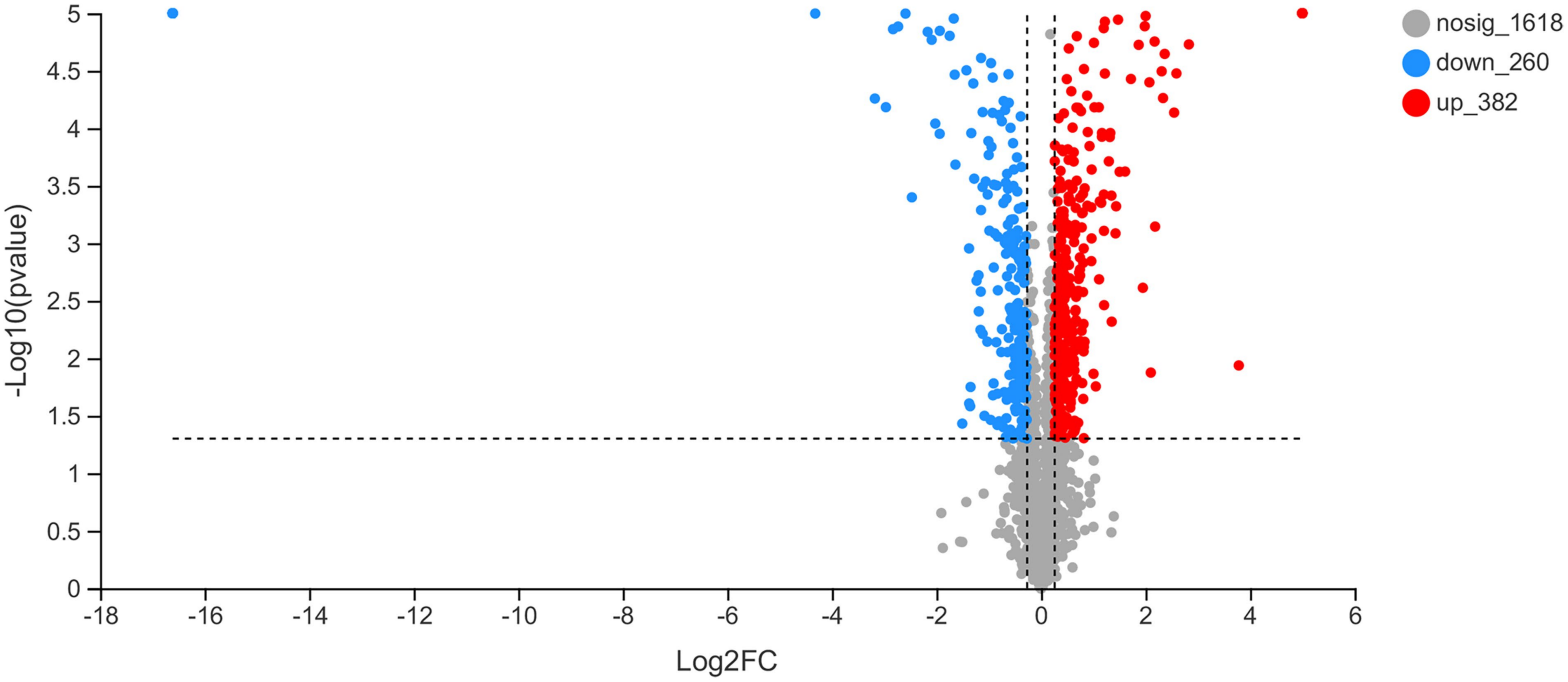
Changes in the proteome of *Escherichia coli* K12 following RcsC deletion. Volcano plots of the 2,260 identified proteins are shown. Red dots represent proteins with fold change values > 1.20. Blue dots represent proteins with fold change values < 0.83 (*p* < 0.05).

To determine the potential mechanisms through which *rcsC* deletion affects plantaricin BM-1 sensitivity in *E. coli*, we detected differentially expressed proteins in different strains. When the changed protein expression fold was 1.20, 2,260 differential proteins were identified (*p* < 0.05, Figure 7). The expression of 642 proteins was altered between *E. coli* JW5917 and K12, including 382 upregulated and 260 downregulated proteins (*p* < 0.05). Subcellular localization analysis of differentially expressed proteins revealed that 94.03% were located in the cytoplasm, with plasma membrane proteins accounting for just 5.13% of all differentially expressed proteins, and extracellular proteins accounting for just 0.84%.

GO functional annotation statistics for differential proteins clarifies the biological processes, cellular components, and molecular functions that proteins are involved in at the functional level. GO functional annotation of the differentially expressed proteins revealed that 539 were labeled as biological processes (BP), among which 82.56% were related to cellular processes and 69.39% were related to metabolic processes (Table 2). In particular, these differentially expressed proteins were significantly enriched for metabolic processes (GO:0008152), cellular processes (GO:0009987), responses to stimuli (GO:0050896), biological regulation (GO:0065007), and localization (GO:0051179). In terms of cellular components (CC), proteins were significantly enriched for protein-containing complexes (GO:0032991) and cellular anatomical entities (GO:0110165). Proteins related to molecular function (MF) were significantly enriched for transporter activity (GO:0005215), catalytic activity (GO:0003824), and binding (GO:0005488).

**Table 2:** GO categories of differentially expressed proteins in *Escherichia coli* JW5917.

Term type	GO term	GO ID	JW5917 vs. K12 down percent	JW5917 vs. K12 up percent
Biological process	Immune system process	GO:0002376	1/260	1/382
Biological process	Carbon utilization	GO:0015976	2/260	0/382
Biological process	Biological regulation	GO:0065007	27/260	50/382
Biological process	Metabolic process	GO:0008152	173/260	201/382
Biological process	Intraspecies interaction	GO:0051703	2/260	0/382
Biological process	Multi-organism process	GO:0051704	1/260	1/382
Biological process	Locomotion	GO:0040011	5/260	4/382
Biological process	Reproductive process	GO:0022414	0/260	5/382
Biological process	Sulfur utilization	GO:0006791	0/260	3/382
Biological process	Cellular process	GO:0009987	194/260	251/382
Biological process	Developmental process	GO:0032502	0/260	3/382
Biological process	Interspecies interaction	GO:0044419	2/260	2/382
Biological process	Localization	GO:0051179	41/260	56/382
Biological process	Biological adhesion	GO:0022610	2/260	3/382
Biological process	Viral process	GO:0016032	0/260	1/382
Biological process	Detoxification	GO:0098754	1/260	4/382
Biological process	Signaling	GO:0023052	1/260	1/382
Biological process	Nitrogen utilization	GO:0019740	1/260	0/382
Biological process	Response to stimulus	GO:0050896	57/260	97/382
Cellular component	Protein-containing complex	GO:0032991	55/260	48/382
Cellular component	Cellular anatomical entity	GO:0110165	197/260	270/382
Molecular function	Translation regulator activity	GO:0045182	1/260	1/382
Molecular function	Transcription regulator activity	GO:0140110	10/260	21/382
Molecular function	Structural molecule activity	GO:0005198	0/260	1/382
Molecular function	ATP-Dependent activity	GO:0140657	10/260	19/382
Molecular function	Cytoskeletal motor activity	GO:0003774	1/260	1/382
Molecular function	Molecular adaptor activity	GO:0060090	1/260	1/382
Molecular function	Protein folding chaperone	GO:0044183	1/260	0/382
Molecular function	Molecular carrier activity	GO:0140104	3/260	2/382
Molecular function	Antioxidant activity	GO:0016209	2/260	9/382
Molecular function	Transporter activity	GO:0005215	41/260	34/382
Molecular function	Molecular function regulator	GO:0098772	1/260	4/382
Molecular function	Small molecule sensor activity	GO:0140299	2/260	1/382
Molecular function	Binding	GO:0005488	179/260	227/382
Molecular function	Molecular transducer activity	GO:0060089	3/260	3/382
Molecular function	Catalytic activity	GO:0003824	183/260	264/382

Next, we performed KEGG functional annotation to verify the functional classification of pathways and the functional roles of the differentially expressed proteins. Crystal violet staining and LSCM revealed that the biofilm content was significantly reduced in *E. coli* JW5917. KEGG and GO analyses screened 17 proteins related to biofilm formation, including 10 downregulated and 7 upregulated proteins (Table 3). In particular, arabinose 5-phosphate isomerase (GutQ), biofilm regulator (BssR), phosphate regulon transcriptional regulatory protein (PhoB), and autoinducer-2 kinase (LsrK) are directly related to biofilm formation, while the N-acetylglucosamine-specific EIICBA component (NagE), maltodextrin phosphorylase (MalP), and RNA polymerase sigma factor (RpoS) can indirectly regulate biofilm formation through pathway regulation or signal responses.

**Table 3:** Differentially expressed proteins related to biofilm formation in *Escherichia coli* JW5917.

Accession number	Description	Fold change	*P*-value	Protein
P30855	Sensor histidine kinase	0.75	0.007079	EvgS
P0ACZ4	DNA-binding transcriptional activator	0.6979	0.008196	EvgA
P17115	Arabinose 5-phosphate isomerase	0.7687	0.01128	GutQ
P13445	RNA polymerase sigma factor	1.742	0.000532	RpoS
P0AAY1	Biofilm regulator	0.5295	0.021	BssR
P77432	Autoinducer-2 kinase	0.7856	0.000475	LsrK
P0AFJ5	Phosphate regulon transcriptional regulatory protein	1.251	0.03157	PhoB
P76237	Probable diguanylate cyclase	1.783	0.007179	DgcJ
P0A9Q1	Aerobic respiration control protein	1.242	0.005906	ArcA
P09323	N-acetylglucosamine-specific EIICBA component	0.638	0.001929	NagE
P04128	Type-1 fimbrial protein	0.3523	0.037	FimA
P69913	Carbon storage regulator	0.3423	0.3935	CsrA
P0AEV1	Regulator of RpoS	1.253	0.001616	RssB
P45578	S-ribosylhomocysteine lyase	1.283	0.000602	LuxS
P00490	Maltodextrin phosphorylase	0.6347	0.0004	MalP
P0ACJ8	cAMP-activated global transcriptional regulator	1.274	0.01337	Crp
P0A9E5	Fumarate and nitrate reduction regulatory protein	0.8142	0.03268	Fnr

### Effects of biofilm-related gene mutations on the sensitivity of *Escherichia coli* to plantaricin BM-1

**Figure 8:**
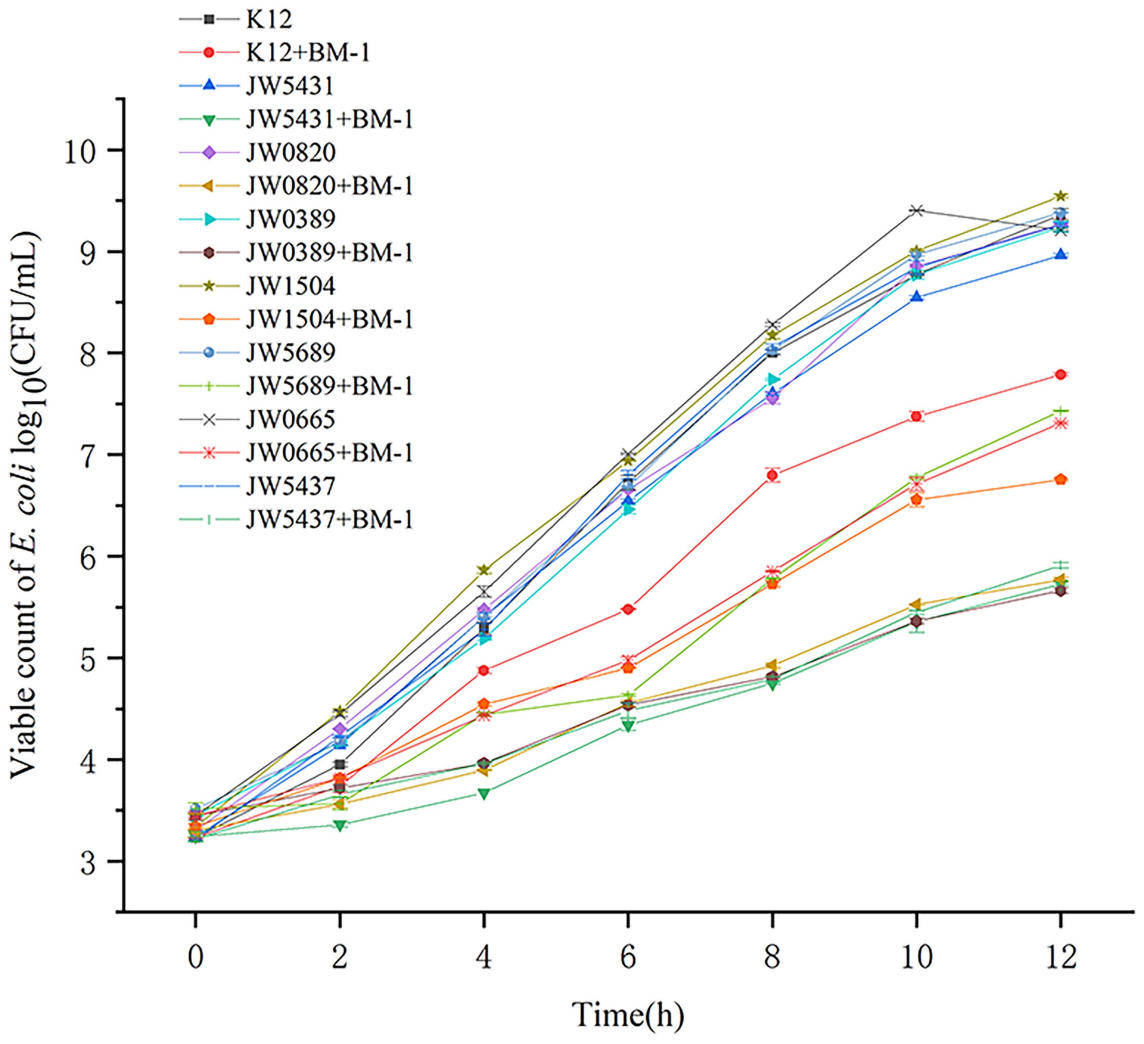
Effects of plantaricin BM-1 on the growth of wild-type *Escherichia coli* K12, mutant *E. coli* JW5431, JW0820, JW0389, JW1504, JW5689, JW0665, and JW5437 strains (*p* < 0.05).

The effect of plantaricin BM-1 on the growth of *gutQ*, *bssR*, *phoB*, *lsrK*, *nagE*, *malP*, and *rpoS E. coli* mutants was assessed by generating standard growth curves (Figure 8). The number of viable *E. coli* JW5431 cells was slightly lower (*p* > 0.05) than that of other mutants, possibly due to a decrease in activity caused by long-term storage. Under the treatment of plantaricin BM-1, *E. coli* JW5431, JW0820, JW0389, JW1504, and JW5437 grew slowly for the first 12 h and the viable cell count only reached 5.00 log_10_ or 6.00 log_10_ CFU/ml, which was significantly lower than that of *E. coli* K12 (*p* < 0.05). However, the sensitivity of *E. coli* JW5689 and JW0665 to plantaricin BM-1 did not differ significantly compared to *E. coli* K12, possibly because not all the proteins associated with biofilm synthesis are associated with sensitivity to plantaricin BM-1. Taken together, these findings suggest that the Rcs phosphorelay could affect the sensitivity of *E. coli* to plantaricin BM-1 by regulating the expression of GutQ, BssR, PhoB, LsrK, and RpoS.

## Discussion

The Rcs phosphorelay is one of the most complex TCSs in *E. coli* K12, with three core components: RcsC, RcsB, and RcsD. This system was originally described as a regulator of colanic acid synthesis (Gottesman and Stout, 1991); however, recent research has shown that the Rcs phosphorelay system also plays a role in acid resistance, cell division, motility, and biofilm formation. The Rcs phosphorelay can be activated by several conditions, including osmotic and acid shock, desiccation, and the perturbation of cell envelope integrity (Francez-Charlot et al., 2003; Clarke, 2010; Wall et al., 2018). In addition, studies have shown that the sensitivity of the Rcs phosphorelay system to lysozyme increases when it is blocked genetically and that the Rcs phosphorelay system can be induced by lysozyme, and encodes two lysozyme inhibitors, Ivy and MliC. The sensitivity of lysozyme can be alleviated by complementation with Ivy and MliC (Callewaert et al., 2009). Previously, we found that approximately 80% of the genes identified as members of the ampicillin regulon in *E. coli* treated with bactericidal levels of ampicillin are also regulated by the Rcs phosphorelay (Kaldalu et al., 2004; Huang et al., 2006). We also found that the deletion of the BasS/R TCS markedly increased sensitivity to plantaricin BM-1. TCSs, such as BasS/R, the Rcs phosphorelay, and PhoQ/P, are often thought to be related to the synthesis and modification of cell surface polysaccharides. Indeed, the absence of BasS/R has been shown to cause abnormal changes in the regulatory networks that exist between these systems (Liu et al., 2022 and our unpublished observations). Although the deletion of RcsC in the Rcs phosphorelay significantly increases the sensitivity of *E. coli* to plantaricin BM-1, the mechanism is unclear. In this study, we found that biofilm formation was significantly reduced in *E. coli* JW5917 compared to *E. coli* K12, with proteomic analysis further revealing that the differentially expressed proteins between these strains were mainly distributed in the cytoplasm and were directly or indirectly involved in biofilm formation. Moreover, we found that the deletion of genes encoding GutQ, BssR, PhoB, LsrK, and RpoS significantly increased the sensitivity of *E. coli* K12 to plantaricin BM-1.

Biofilms are organized bacterial populations encapsulated in a bacterial extracellular polymeric substance (EPS) matrix that can adhere to each other on biotic or abiotic surfaces (Rather et al., 2021). EPS mainly consists of polysaccharides, but other biomolecules like proteins, lipids, and nucleic acids are also present in EPS (Cortes et al., 2011; Gupta et al., 2016). Studies have shown that biofilm formation contributes toward the development of antibiotic resistance and the formation of persistent cells that are responsible for untreatable microbial infections (Pang et al., 2019; Rather et al., 2021). Previous studies have found that TCSs can achieve antibiotic resistance by regulating biofilm formation. For instance, the GacS/A TCS can participate in the formation of *P. aeruginosa* biofilms and confer resistance to aminoglycosides, such as amikacin and gentamicin (Brinkman et al., 2001). In addition, GacS/A is active in biofilms formed by *Staphylococcus aureus* and confers resistance to antibiotics such as vancomycin (Fridman et al., 2013). In this study, we found that arabinose 5-phosphate isomerase (GutQ), a precursor of the cell envelope lipopolysaccharide component 2-keto-3-deoxy-octulosonate (KDO) (Lim and Cohen, 1966), was downregulated by 0.76-fold in the rcsC mutant. It has been reported that GutQ is involved in biofilm formation, with mutants lacking *gutQ* showing a marked reduction in biofilm formation and increased *gutQ* expression increasing biofilm formation (Herzberg et al., 2006). GutQ expression also correlates negatively with the expression of YdgG, which can affect resistance to various antimicrobials, including crystal violet and streptomycin (Herzberg et al., 2006). BssR is a biofilm regulator that is transcribed during biofilm formation and can regulate biofilm formation through signal secretion (Ren et al., 2004). LsrK, a kinase that can phosphorylate the quorum-sensing auto-inducible molecule AI-2, was also downregulated by 0.78-fold in the *rcsC* mutant. Interestingly, the *lsrK* mutant had a different biofilm structure compared to the wild-type and LsrK has been reported to regulate the expression of biofilm-related genes (Li et al., 2007). Studies have also shown that LsrK may be a potential drug target for solving antibiotic resistance (Linciano et al., 2020); therefore, our findings may provide insights into the use of plantaricin BM-1 as a target in Gram-negative bacteria. Similarly, phosphate regulon transcriptional regulatory protein (PhoB), a dual transcriptional regulator that activates the expression of Pho regulators in response to environmental phosphate, was upregulated by 1.25-fold in our proteomics analysis. Studies have shown that *E. coli* PhoB can be activated under unrestricted phosphate conditions to inhibit biofilm formation (Grillo-Puertas et al., 2016). As the passive response regulator of the PhoR/B TCS, PhoB remains active by default and requires the interference of environmental signals to shut down the system. However, our proteomic analyses revealed no significant changes in PhoR expression. RNA polymerase factor sigma (RpoS) is an RNA polymerase subunit that acts as a master regulator of the general stress response in *E. coli* (Hengge-Aronis, 2002; Weber et al., 2005) and is regulated at the levels of protein degradation, transcription, translation, and activity. Studies have shown that *rpoS* regulates the formation of *E. coli* cell membranes (Adnan et al., 2010) and that high *rpoS* expression inhibits *E. coli* biofilm formation (Corona-Izquierdo and Membrillo-Hernandez, 2002). Moreover, it has been reported that *rpoS* is significantly upregulated after treatment with ampicillin and mitomycin C (Dapa et al., 2017; Mohiuddin et al., 2022), consistent with our findings after treatment with plantaricin BM-1.

In summary, our study demonstrates that decreased Rcs phosphorelay expression can increase the sensitivity of *E. coli* K12 to plantaricin BM-1. However, we found that the sensitivity of *E. coli* K12 to plantaricin BM-1 was not altered after RcsB and RcsD deletion, which may indicate that biofilm formation is only reduced after *rcsC* mutation. Thus, RcsC may can control the effect of the Rcs phosphorelay on biofilm formation. Another possibility is that because RcsC itself contains both His and Asp., the mechanism regulating the sensitivity of *E. coli* K12 to plantaricin BM-1 may be completed through this His-Asp process (Wall et al., 2018). In conclusion, mutations in the Rcs phosphorelay resulted in significantly reduced biofilm formation in *E. coli*, resulting in reduced cell resistance to stress and affecting the sensitivity of *E. coli* K12 to plantaricin BM-1. In future studies, we will attempt to construct an *rcsCDB* three-gene mutant to further verify the mechanism through which the Rcs phosphorelay regulates the sensitivity of *E. coli* K12 to plantaricin BM-1.

## Data availability statement

The datasets presented in this study can be found in online repositories. The names of the repository/repositories and accession number(s) can be found at: http://www.proteomexchange.org/, PXD037354.

## Author contributions

ZB conceived and designed the experiments, performed the experiments, and analyzed the data. WL analyzed the data. JJ, LJ, and YH contributed materials. HZ conceptualization and methodology. YX conceived and designed the experiments. All authors contributed to the article and approved the submitted version.

## Funding

This work was supported by the Research project of Beijing Municipal Commission of Education (KM201810020016).

## Conflict of interest

The authors declare that the research was conducted in the absence of any commercial or financial relationships that could be construed as a potential conflict of interest.

## Publisher’s note

All claims expressed in this article are solely those of the authors and do not necessarily represent those of their affiliated organizations, or those of the publisher, the editors and the reviewers. Any product that may be evaluated in this article, or claim that may be made by its manufacturer, is not guaranteed or endorsed by the publisher.
